# The Otoacoustic Emissions in the Universal Neonatal Hearing Screening: An Update on Selected Asian States (2005 to 2025)

**DOI:** 10.3390/children13010060

**Published:** 2025-12-31

**Authors:** Stavros Hatzopoulos, Ludovica Cardinali, Piotr Henryk Skarzynski, Giovanna Zimatore

**Affiliations:** 1 Clinic of Audiology & ENT, University of Ferrara, 44122 Ferrara, Italy; 2 Department of Life Science, Health, and Health Professions, Link Campus University, 00165 Rome, Italy; l.cardinali@unilink.it (L.C.); giovanna.zimatore@uniecampus.it (G.Z.); 3 Heart Failure and Cardiac Rehabilitation Department, Faculty of Medicine and Dentistry, Medical University of Warsaw, 02-005 Warsaw, Poland; p.skarzynski@inz.waw.pl; 4 Institute of Sensory Organs, 05-830 Nadarzyn, Poland; 5 World Hearing Center, Department of Teleaudiology and Screening, Institute of Physiology and Pathology of Hearing, 02-042 Warsaw, Poland; 6 Department of Theoretical and Applied Sciences Applied Physics, eCampus University, 22060 Novedrate, Italy

**Keywords:** Asia, congenital hearing loss, newborn hearing screening, otoacoustic emissions, well babies, NICU, bilateral hearing loss

## Abstract

**Background**: Although significant progress has been made in Neonatal Hearing Screening (NHS) over the past two decades, the available data on Universal Neonatal Hearing Screening (UNHS) practices across Asia remain limited. The aim of this scoping review was therefore twofold: (a) to identify and synthesize the most recent literature (within the past 20 years) concerning NHS/UNHS programs in Asian states, and (b) to summarize evidence on screening procedures, the intervention strategies, and the estimated prevalence of congenital hearing loss (HL), with particular attention to cases of bilateral impairment. **Methods**: In line with previous reports from our group on the screening practices in Europe and in Africa, queries were conducted via the PubMed, Scopus and Google Scholar databases for the time window of 2005–2025. The Mesh terms used were “Otoacoustic Emissions (OAE)”, “Universal Neonatal Hearing Screening”, “congenital hearing loss”, “well babies” and “ASIA”, as well as all 50 Asian state names. Only research articles and review papers were considered as good candidates. The standard English language filter was used. **Results**: To maintain homogeneity in terms of state area and population, the studies conducted in China and India were excluded from this report and will be the focus of a dedicated paper. Data from 31 papers were considered, reflecting the neonatal hearing practices of 17 Asian states, of which in 12, UNHS programs are considered mandatory. **Conclusions**: The information on the Asian NHS is limited to a low percentage of Asian states. The available data strongly suggest that audiologists and other hearing professionals, involved in regional or national screening initiatives, should collect systematically and disseminate the screening information through peer-reviewed scientific publications. The latter will contribute to a broader understanding of program effectiveness and will facilitate international benchmarking.

## 1. Introduction

The goal of any Neonatal Hearing Screening (NHS), or of an Early Hearing Detection and Intervention (EHDI) protocol, is to identify infants who may present sensorineural and conductive hearing impairments. Data from the literature clearly suggest that language development and the relative development of social and cognitive abilities depend on the early identification and rehabilitation of hearing impairments [1,2]. Universal NHS (UNHS) can now be performed using two assessment protocols, based on Otoacoustic Emissions (OAEs) and the Automated Auditory Brainstem Responses (AABRs) [3,4]. Previously reported data in the literature provide the essential information regarding the effects of hearing screening on language development and the inner workings of the now-established clinical screening techniques [5].

OAEs are a rapid, non-invasive, and economical method. Certain brief stimuli (such as clicks, chirps, and tone bursts) are produced by microphones in a tiny probe, which is placed into the newborn’s ear canal, and they evoked responses from the cochlea, recorded by the same microphones. Applications for this type of technology are numerous, and they extend as well into the adult population, including the detection of noise-induced hearing loss (HL) or age-related hearing impairment [6]. Most importantly, the data in the literature suggest that OAE hearing screening protocols can detect hearing impairment significantly earlier than the traditional pure tone audiometry assessment [7].

As we have previously reported in the analysis of the NHS European [8] and African data [9,10], NHS programs appear to encounter numerous implementation challenges, even though hearing screening is regarded as a necessary clinical practice.

This scoping review focuses on the NHS practices of a number of Asian countries and follows the guidelines we have established in the previous publications. The present work addresses five key questions: (i) Which states have implemented UNHS programs? (ii) What proportion of newborns are screened? (iii) What is the prevalence of congenital, particularly the bilateral, hearing loss (HL)? (iv) If reported, what are the follow-up rates? and (v) Which screening protocols and OAE technologies are most commonly employed?

## 2. Materials and Methods

We sourced scientific articles using the PubMed, Scopus, and Google Scholar search engines. As in the previous reviews, we have selected a 20-year time window (2005–2025) to address existing informational gaps. Baseline data were derived from the comprehensive, survey-based study by Neumann [11] in 2020, which analyzed healthcare systems across 196 states worldwide.

Asia accounted for approximately 4.8 billion inhabitants in 2024 [12], encompassing countries with markedly diverse demographic, healthcare, and regulatory characteristics. For methodological reasons, studies conducted in China and India were excluded from the present review. These two countries represent exceptional cases within the Asian region, owing to their extremely large populations, highly heterogeneous healthcare infrastructures, and the long-standing implementation of independently developed newborn hearing screening programs. Inclusion of China and India would have disproportionately influenced the synthesis and limited the comparability of findings across countries with smaller populations and more homogeneous regulatory frameworks. The present review, therefore, focuses on Asian countries with broadly comparable programmatic and contextual characteristics, while evidence from China and India will be addressed in a separate forthcoming manuscript. The assessed Asian states (in bold) as shown in Table A1 in Appendix A.

The search of the literature was carried out in September 2025 in accordance with the PRISMA 2020 framework (website accessed 4 July 2025, the check list is reported in Supplementary Materials). Three primary MeSH terms guided the selection process: “OAE,” “Neonatal Hearing Screening,” “congenital hearing loss,” and “Asia” or the name of each Asian state. Only English-language research articles were included (reviews and single or sporadic case reports were not considered), while studies dealing with NHS outside Asia were excluded.

Selection quality was ensured by prioritizing publications in peer-reviewed journals. Preference was also given to studies presenting transparent methodologies and a consistent application of established screening protocols.

The inclusion criteria for the review were established as follows: (i) the geographical affiliation of the study (Asian origin versus other regions); (ii) the size of the screened population, with preference given to larger cohorts; and (iii) the recency of data, selecting the most up-to-date study or studies available for each country.

**Figure 1:**
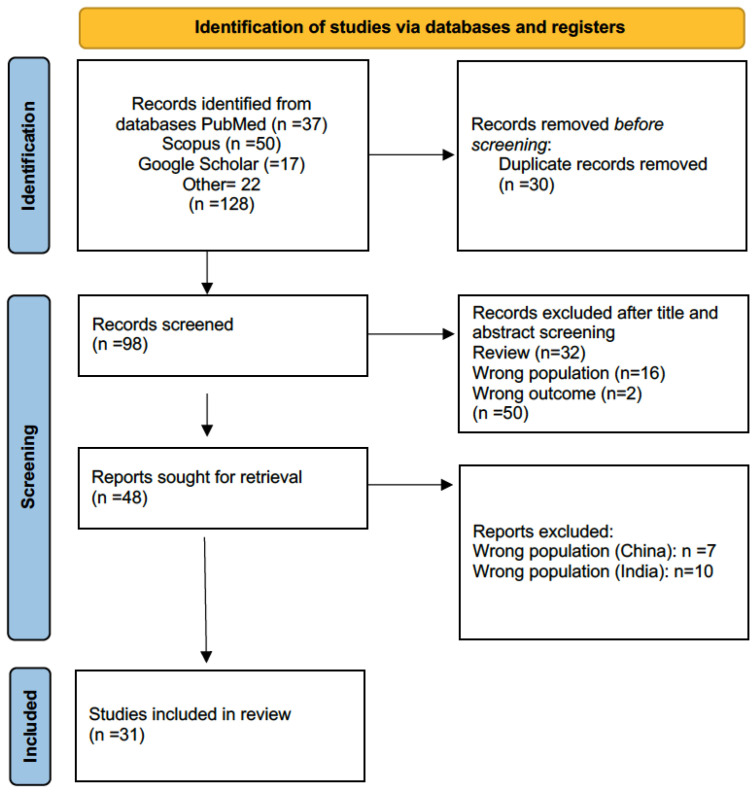
Flow diagram of literature search, according to PRISMA criteria (https://www.prisma-statement.org/prisma-2020, accessed 4 July 2025), with the steps followed in the manuscript selection procedure. After the application of the selection criteria, the initial 128 manuscripts were reduced to 31.

References were drawn from PubMed, Scopus, and Google Scholar. Two independent reviewers assessed the retrieved material, resulting in a final selection of 48 eligible papers. A second screening excluded 17 papers for geographic origin in China and India. The PRISMA flow diagram is presented in Figure 1, while the studies included in the review are summarized in Table 1.

## 3. Results

The data extracted from the 31 selected studies were classified alphabetically according to the state of origin, and the information obtained is summarized in Table 2.

### 3.1. Indonesia

Zislavsky et al. [44] coordinated a multicenter study in Indonesia on the factors associated with the time of diagnosis and habilitation of congenital HL. They reported in 2023 that the estimated prevalence of congenital HL in Indonesia was a high value of approximately 110/1000, showing an increasing pattern from the reported value of 80/1000 in 2013.

In terms of NHS, a prospective study conducted by Falerina et al. [13] at Dr. Soetomo General Academic Hospital in Surabaya evaluated the feasibility and outcomes of newborn hearing screening using Distortion Product OAEs (DPOAEs). Although no national UNHS policy was in place (in the year 2023), this investigation represents one of the few structured implementations of serial hearing screening in the country, with the downside of a rather small sample size. A total of 60 neonates were enrolled, equally divided between those with (n = 30) and without (n = 30) risk factors for HL. Screening was conducted universally within the hospital setting, using DPOAEs at three time points: >24 h after birth, 1 month, and 2 months of age. Infants who continued to yield REFER results after the third session were referred for Auditory Brainstem Response (ABR) testing. Across the three sequential screenings, the proportion of infants screened reached 100% of those born in the study hospital. The pass rate improved markedly from 18% at >24 h, to 36% at 1 month, and 96% at 2 months, demonstrating the effectiveness of serial screening in reducing false positives. Only two infants (3.3%), one with prematurity and one with low birth weight (LBW), retained REFER outcomes after the final screening and were referred for confirmatory ABR evaluation. This represents an apparent prevalence of suspected congenital HL of approximately 3% (a rather high estimate), although no confirmed ABR results were reported.

Follow-up adherence across the three test sessions was excellent, with all 60 newborns completing the screening sequence. The study highlights that DPOAE technology is feasible, cost-effective, and adaptable to resource-limited Indonesian hospital settings. The authors recommend serial universal screening at birth, 1 month, and 2 months to reduce unnecessary referrals and ensure early detection before 6 months of age.

### 3.2. Iran

Saki et al. [14] evaluated 92,521 newborns (46,147 boys and 46,374 girls) between March 2013 and April 2016, from the Khuzestan Province (southwestern Iran) as part of a national UNHS initiative, covering virtually 100% of eligible live births in the region (67,780 urban; 24,741 rural). A two-stage protocol combining Transient-Evoked OAEs (TEOAEs) and Automated ABR was used: first within 48 h after birth, repeated at ≤1 month, and with full diagnostic ABR/OAE evaluation by ≤3 months for persistent REFER cases. The coverage rate approached 100%, with a 4.2% loss to follow-up (LTF).

Confirmed HL was diagnosed in 223 infants, corresponding to a prevalence of 2.41 per 1000 live births. Of these, 141 infants (1.52 per 1000) presented bilateral HL and 82 infants (0.89 per 1000) unilateral impairment. Most cases (87%) were sensorineural, 12.5% were auditory neuropathy, and a small minority were conductive losses (5%). Severe-to-profound degrees of loss predominated, with no significant sex difference (*p* = 0.29). Of the 10,804 neonates who required rescreening, 4.23% (457 infants) were lost to follow-up. Among those who completed the second stage, 98.75% of all screened newborns ultimately passed, leaving 1.25% (1161) referred for full diagnostic evaluation. Follow-up compliance was generally high, though slightly lower in rural settings due to limited audiological resources and travel barriers. The combined TEOAE + AABR strategy effectively minimized false positives and captured auditory neuropathy cases, providing outcomes comparable to high-income states.

### 3.3. Israel

Israel has fully implemented a UNHS program, mandated by the Ministry of Health since January 2010. The program requires that all newborns be screened before hospital discharge, reflecting one of the most comprehensive and well-organized early hearing detection systems in the Middle East. The program combines OAE testing and ABR to ensure early detection of congenital and neural hearing disorders, including auditory neuropathy. Three complementary studies described the national screening outcomes in Israel.

Gilbey et al. [15] analyzed 5496 live births at Ziv Medical Center (Zefat). A two-stage TEOAE + AABR protocol achieved 94.8% coverage, 5.2% referral, and 70–81% diagnostic follow-up by the age of three months. In total, 34 infants were diagnosed with hearing impairment (27 conductive, 7 mild–moderate sensorineural).

Ziv et al. [16] examined 238 children diagnosed with hearing impairment post-UNHS. Among those born from consanguineous unions, bilateral HL and delayed or incomplete compliance with cochlear implantation were significantly more frequent. The authors of the paper comment that Israeli UNHS programs reach a >95% national coverage by applying TEOAE + AABR protocols, though sociocultural factors such as consanguinity still affect long-term outcomes and follow-up compliance.

In 2025, Farladansky-Gershnabel et al. [17] reported data from 5621 infants with normal hearing, identifying 6.5% false-positive OAE results. Cesarean section and vacuum-assisted deliveries were significant predictors of false-positive outcomes (OR ≈ 7–15 vs. vaginal delivery), underscoring the influence of perinatal factors on screening accuracy.

### 3.4. Japan

The first pilot NHS study was conducted in the Okayama prefecture from 2001 to 2005, and Fukushima et al. [18] reported the data in 2008. According to the authors, 47,346 infants (corresponding to a coverage of 95%) were screened with AABR and followed for 24 months. From this sample, 248 neonates presented a hearing impairment (108 bilateral HL and 140 unilateral HL). Three cases who initially passed the AABR test were later (the time is not specified) diagnosed with a late-onset HL. The authors also comment that the screening protocol of the pilot study was carefully designed to work with the Japanese societal trends (not specified), suggesting that societal factors are equally important in NHS strategies to the actual screening protocols selected.

In 2020, Sato et al. [19] published data from the Akita region, using a time frame of 5 years and achieving the second largest assessed NHS sample in Japan. From 2012 to 2016, 35,461 infants were screened via a two-stage AABR, resulting in a coverage rate of 94,7%. The AABR protocol was the same as in the Fukushima et al. study, where a two-stage AABR was used to identify HL. REFER cases from the first AABR were retested one day before discharge. The REFER cases from the second stage were diagnosed with a clinical ABR within a timeframe of 3 months. The authors reported that 179 infants were identified with a probable HL and were sent to the otorhinolaryngology department of the collaborating hospitals for a final diagnosis. Of those, 36 were not included in the assessment. The identified HL cases included 47 with bilateral deficits, 55 with a right-ear and 42 with a left-ear HL. The overall incidence of HL reported was 144/33,545 = 4.3/1000. Genetic testing was also conducted on the bilateral HL cases to identify the possible causes of the congenital HL. Only one infant was identified with a mutation of the Cap-junction beta-2 protein.

### 3.5. Jordan

Recent studies in Jordan have focused not only on screening implementation but also on identifying key genetic and demographic determinants of congenital and early childhood HL.

The most recent and comprehensive data come from Qanawati et al. [20], who investigated the relationship between consanguineous marriage and HL in Jordanian children. The authors conducted a retrospective analysis between 2020 and 2023 of 416 pediatric patients (<18 years) at the Al-Ahliyya Amman University Hearing and Speech Center. Screening and diagnostic methods included DPOAEs, ABR, and pure-tone audiometry, indicating adherence to established early detection protocols. In the analyzed cohort, 77.9% (n = 324) of children were diagnosed with HL, with a predominance of sensorineural HL (SNHL; 65%), followed by conductive (33%) and mixed (1%) types. The mean age of onset was 5 months, reflecting early detection consistent with neonatal or infantile origin. While the study did not explicitly differentiate between congenital and acquired etiologies, the findings underscore a high burden of early-onset and likely genetic HL within the Jordanian pediatric population. Logistic regression revealed that children from consanguineous marriages had significantly higher odds of HL (log odds = 0.895; *p* = 0.029), particularly for sensorineural and mixed types, and that family history acted as a compounding factor (interaction term *p* = 0.007).

### 3.6. Malaysia

Abdullah et al. [21] reported HL data from neonatal residents of the Intensive Care Unit (NICU) in a Kuala-Lampur tertiary hospital. The authors assessed data from 2713 infants from January 2014 to December 2016. The tested protocol consisted of OAEs and AABR (the type of OAEs was not specified). 214 Infants who resulted as REFER by both screening procedures were tested by a clinical ABR within a time frame of 30–90 days. From those, the final assessment was possible only for 105, and in this group, 40 neonates were diagnosed with a HL. In total, 32 presented a bilateral HL, 7 presented a right-ear unilateral HL and 1 presented a left-ear unilateral HL. The estimated incidence of HL was 40/(2713-109 who did not complete the final ABR) = 15.36/1000, a rather high value. From the considered risk factors only, craniofacial anomalies were found to be significantly related to HL. For infants presenting three or more risk factors, the probability of having a hearing impairment was 100%.

Wong et al. [22] reported the results of a retrospective analysis on UNHS data from four public Malaysian hospitals in the time frame of 2015 and 2016. The authors have presented their data in terms of each year to show the progress achieved from one year to another. In this review, the data have been pooled together. A total of 58,862 infants were assessed with a mean regional coverage of 81.2%. The screening protocol consisted of a first stage conducted with DPOAEs and the second with AABR. According to the authors, “Depending on hospital resources, automated AABR and DPOAE were used, either alone or combined, as the hearing screening tool”. The total number of infants diagnosed with a hearing impairment was 186, suggesting an HL incidence of 3.15/1000. In terms of quality measures, the study data showed that “ The return for follow-up rates for each participating hospital was generally below the recommended benchmark of 95%” [45]. The mean age at screening was 3.9 days and 3.3 days, respectively, for the years 2015 and 2016, showing an improvement in the age of identification. From the 186 infants who presented HL, the mean age for the 70 infants diagnosed with a permanent HL was 4.7 ± 0.7 months in 2015 and 3.6 ± 0.9 months in 2016, both below the expected 6-month intervention limit.

### 3.7. Nepal

The study by Shrestha et al. [23] at Dhulikhel Hospital, Kathmandu University Hospital, represents the first large-scale prospective implementation of UNHS in Nepal.

The Dhulikhel Hospital program began in February 2017 as a structured pilot of universal screening for all inborn infants and those admitted to the Neonatal Intensive Care Unit (NICU). The study demonstrated that a two-stage Automated ABR protocol can be successfully implemented. During the study period (February 2017–October 2019), 5517 of 5956 live births (92.6%) were screened. Of these, 422 (7.7%) were NICU infants and 5095 (92.3%) were well babies. In the first-stage screening, 30% of well babies and 35% of NICU babies were referred (“REFER”). In the second-stage rescreening (after 6 weeks), referral rates dropped to 6.6% among well babies and 9.0% among NICU babies. The overall referral rate after the second screening was 6.7% (374/5517), demonstrating the value of a two-step process in reducing false-positive results. The prevalence of confirmed congenital HL was not directly reported, as diagnostic follow-up ABR outcomes were beyond the study’s scope. However, using referral as a proxy indicator, the apparent prevalence of suspected congenital HL was approximately 6.7% overall and higher in NICU infants (9%) compared to well babies (6.6%). Among the risk factors analyzed, LBW and prematurity were the most frequent, followed by ototoxic drug exposure, hyperbilirubinemia, and mechanical ventilation. Follow-up compliance was high: all infants who failed the first screening were scheduled for rescreening at 6 weeks, coinciding with immunization visits. The integration with vaccination schedules minimized attrition, resulting in nearly complete follow-up across both well and sick newborns. The authors emphasized that this combined health-service approach is feasible and sustainable for national scale-up.

### 3.8. Oman

Abdullah et al. [24] reported data from a retrospective review conducted from January 2016 to December 2018 in a tertiary hospital in Oman. A sample of 12,743 infants was assessed, and the reported regional coverage was 90%, dropping to 88.4% for the second screening stage. The testing protocol consisted of a two-stage TEOAEs followed by a clinical ABR for the resulting REFER cases. In total, 43 infants were diagnosed with HL, 29 presented a bilateral HL and 11 presented with unilateral losses. The estimated HL incidence for this sample was 43/12,743 = 3.37/1000. In terms of risk factors, significant relationships with HL were found for craniofacial anomalies and syndromic illness. The authors reported relatively long times for the final HL confirmation for the two TEOAE stages as 7.98 and 17.3 weeks, respectively.

For 13 infants of the 43 identified with HL, some sort of early intervention strategy was applied (the authors mention amplification, probably intending the use of hearing aids). The paper also examined the factors which have caused a low return rate, and they commented, “More than 60% of the participants felt that the long waiting period and/or delay in obtaining appointments were the key reasons for the delay in receiving early intervention, and a significant number of them missed appointments as they were not reminded on time”. The latter clearly suggests that emerging UNHS programs need a very systematic patient administration, especially after the initial HL identification (intervention phase)

### 3.9. Pakistan

Pyarali et al. [25] conducted a small prospective cross-sectional study in a Karachi hospital, from November 2020 to April 2021, assessing all born infants in that timeframe. 267 infants were tested with a double-stage OAE protocol (not specified by the authors). In total, 18 infants resulted in REFER and were evaluated by a clinical ABR. Of those, 10 were found with a bilateral HL and 8 with a unilateral HL. For the 42 NICU residents, the following risk factors were identified as important contributors to the observed HL: ototoxic medications (by the mother), radiation exposure (of the mother), permanence in the NICU (days), gestational age and low APGAR score.

The authors make a series of interesting comments regarding the crucial elements of UNHS: (i) they describe their poor experience with LTF (Loss to Follow) for 10 infants, who were identified with a bilateral HL, where only 3 returned for the additional medical checking. Additionally, they discuss some other important factors related to LTF (who were also encountered in a number of African states [9]) in the following: “It is important to discuss with the parents, their failure to understand the importance of screening as well as the early intervention and the need for collaboration between parents and service providers. Other reasons included the lack of transport as they were living in small villages and cities, lack of interest as the family was not prepared for further testing because of financial issues, lack of awareness about early HL identification, social and cultural stigma related to deafness, and negative parental attitude towards hearing screening protocol”.

### 3.10. Philippines

In 2016, Labra et al. [26] conducted a survey on the implementation of UNHS in the Philippines, across 51 newborn hearing screening centers in 12 regions, to assess their compliance with screening protocols, staffing, IT capabilities, and access to specialists. Unfortunately, the paper does not provide any “raw numbers” in terms of the actual infants screened and who and how many have received a hearing intervention. Although important data is missing, the paper provides useful peripheral information on the UNHS practices in the Philippines. The data reported can be grouped into the following:

- From the available centers, 96.1% had certified personnel.
- Only 41.67% of surveyed regions had centers offering confirmatory testing and early intervention services.
- Access to specialists was limited, with only 39.2% of centers having clinical audiologists and 23.5% having speech-language pathologists.
- Assessment was performed initially via a two-stage OAE protocol and then by a clinical ABR/ASSR to verify infants with a suspected hearing deficit.
- Data management was a major issue, with only 3.9% of centers using the online registry and 5.9% using registry cards.
- Internet access was available in 58.8% of centers, but data submission methods were largely non-compliant.
- Patient follow-up systems were inadequate, with only 27.5% of centers tracking newborns referred for confirmatory testing.

The study highlights deficiencies in confirmatory testing, early intervention services, and data management, emphasizing the need for regional centers, improved IT systems, and better resource allocation. Recommendations include decentralizing program management, streamlining registry card procurement, enhancing training and certification processes, and involving pediatricians and community health workers in the program.

### 3.11. Saudi Arabian Emirates

Three studies published between 2005 and 2025 describe the gradual development and refinement of the UNHS programs in Saudi Arabia and demonstrate the country’s transition from early hospital-based initiatives to a near-comprehensive national screening system.

Habib & Abdelgaffar [27] conducted one of the earliest prospective NHS studies at King Fahd Hospital, Jeddah. A total of 11,986 newborns were screened using a two-stage TEOAE protocol performed within 48 h of birth and repeated at 5 days of life for initial REFER cases. At the first screening, 91.3% of newborns passed bilaterally, while 8.7% (n = 1042) were referred for repeat testing. After the second-stage OAE, 0.62% (n = 74) remained REFER. Diagnostic ABR testing at 5 months confirmed 22 cases of SNHL (0.18%), of which 20 were bilateral (0.17%) and 2 unilateral (0.02%). The false-positive rate was reduced from 8.7% to 0.62% after the two-stage design. Parental compliance with follow-up reached 95%, reflecting high acceptance of screening. The authors concluded that the TEOAE-based two-stage program was feasible, accurate, and cost-effective, suitable for integration into public health hospitals nationwide.

Alanazi et al. [28] reported a retrospective cross-sectional descriptive study of national UNHS program data collected from multiple hospitals across Saudi Arabia between 2016 and 2019. Their study described the outcomes of an expanded national-level implementation that utilized automated OAE and AABR technologies in parallel, depending on institutional availability. Across all regions, screening coverage exceeded 90% of live births, with an overall referral rate of 2.8% after the first test and 1.1% after rescreening. The prevalence of confirmed congenital HL was approximately 1.8 per 1000 live births, comparable to global averages. The authors reported persistent challenges related to loss to follow-up cases, averaging 10–12% nationally and recommended the digital centralization of screening data and mandatory follow-up pathways to improve coverage consistency across regions. The findings confirmed that Saudi Arabia’s national screening outcomes parallel the data from European and North American programs [8,11,24], reflecting substantial progress in the NHS infrastructure and early diagnosis.

Elbeltagy et al. [29] performed a cross-sectional follow-up study assessing 130 children aged 6–7 years who had previously passed newborn hearing screening. Using pure-tone audiometry (PTA), tympanometry, and TEOAEs, the authors identified seven children (5.4%) with delayed-onset bilateral SNHL. Parental consanguinity was present in 71% of the affected group versus 34% in the control group, representing a statistically significant association (*p* = 0.026, OR = 2.6). None of the affected children had previously experienced otitis media or ototoxic exposure, suggesting a predominantly genetic etiology. The study highlights the importance of post-UNHS surveillance for delayed onset and progressive forms of HL, particularly in populations with high consanguinity rates. The authors recommend periodic audiological screening during early school years to capture late-onset SNHL cases that escape neonatal detection.

The combined information from these studies suggests that Saudi Arabia has developed one of the most comprehensive UNHS infrastructures in the Middle East. The two-stage TEOAE protocol remains the foundational approach, now complemented by AABR confirmation and digital reporting. The prevalence of congenital bilateral HL has stabilized around 0.17–0.18%, while delayed-onset HL affects an additional 5% of school-aged children. The primary etiological factor remains genetic consanguinity, highlighting the need for continued family counseling, long-term follow-up, and the inclusion of molecular genetic testing in national EHDI protocols.

### 3.12. Singapore

Jayagobi et al. [30] reported data on the screening of NICU residents in a tertiary care center, from April 2002 to December 2009. 2906 NICU infants were assessed with an OAE-ABR protocol, and the data were registered within the HITRACK management system. The average age of diagnosis was reported as 4.5 months. Of the 92 infants presenting with HL, 89 had multiple risk factors. The overall incidence of HL for any type and severity was found to be 35/1000, and the rate of congenital permanent HL was 15.4/1000. During additional tests on the infants who passed the first screening after 3–6 months, 12 infants were identified with a permanent late-onset HL, modifying the HL incidence to 19.9/1000. The auditory neuropathy incidence was estimated as 1.1/1000.

Tang et al. [31] presented data from a UNHS program within a time frame of 10 years. A sample of 29,972 infants was assessed with TEOAEs and AABR; 301 infants were found to be non-eligible (deceased or transferred to another hospital). NICU residents were tested with both protocols. A total of 157 infants were referred to a clinical ABR evaluation, but due to incomplete data, only 147 REFER cases were included in the study. Of those, 117 were found with HL. The estimated incidence of HL was reported as 117/29,671 = 3.94/1000. In terms of intervention policies, only 74 infants received assistance, raising, unfortunately, the loss to follow-up value to 35% (41/117). For two cases, the families refused any post-screening intervention.

### 3.13. South Korea

Two key studies conducted in South Korea have critically examined the performance and limitations of UNHS, particularly in detecting delayed-onset or genetically mediated forms of HL. Both investigations highlighted that conventional screening methods, based on OAEs and AABR, may fail to identify a subset of infants who develop significant hearing impairment later in childhood.

Kim et al. [32] evaluated 43 patients with sensorineural HL carrying biallelic SLC26A4 mutations, a genetic defect associated with Pendred syndrome and enlarged vestibular aqueduct (EVA). In total, 14 of these children had undergone newborn hearing screening. Among them, four infants (28.6%) passed the screening bilaterally, while six (42.9%) passed in one ear and failed in the other. Only four were correctly identified as REFER in both ears. For comparison, among 15 children without SLC26A4 mutations, only 2 (13.3%) passed the screening bilaterally. The average age at which bilateral HL was confirmed in the PASS group was approximately 31.5 months, compared with only 1.7 months in those who had been identified as REFER at birth. This significant diagnostic delay underscored the limitation of the OAE- and the AABR-based screening in detecting infants predisposed to progressive or late-onset HL due to genetic causes. The authors concluded that universal newborn hearing screening alone is insufficient in such cases and recommended the addition of molecular genetic testing, particularly for SLC26A4, to improve early identification of at-risk infants.

Lee et al. [33] further examined the clinical implications of these findings by analyzing a cohort of children who had undergone cochlear implantation after initially passing newborn screening. Among 109 children with available NHS data, approximately 22% had passed the screening at birth but subsequently developed profound bilateral HL necessitating cochlear implantation. In these children, the mean age at diagnosis of HL was 28 months, significantly later than the average of three months in those who had failed the neonatal screen. The study confirmed that progressive or late-onset HL is not uncommon among children who initially PASS NHS, especially those with genetic etiologies such as SLC26A4 or GJB2 mutations. The authors emphasized that current screening protocols cannot detect these cases and advocated for post-neonatal audiological surveillance to ensure early diagnosis and intervention.

The combination of the data derived from these two studies demonstrates that while the national UNHS system successfully identifies most congenital and bilateral cases, it fails to detect a proportion of infants with genetically determined, progressive forms of HL. The evidence suggests that integrating genetic testing with newborn screening and establishing longitudinal audiological monitoring could markedly enhance early detection and improve long-term auditory and language outcomes in this population.

### 3.14. Syria

There are no reported studies in the literature on any NHS program in Syria, but a Turkish study by Kaplama et al. [34] in the city of Sanliurfa (close to the Syrian border) reports data on a comparison between Syrian (refugees) and Turkish newborns. Considering the absence of other relevant information on the Syrian NHS, it was decided to include this paper in order to present the NHS characteristics of the neonatal Syrian population (born in Turkey).

From January 2018 to December 2018, 6034 Syrian infants were assessed with a two-stage TEOAE protocol followed, if necessary, by an AABR and, in case of another REFER, by a clinical ABR. 34 infants were admitted to a clinical ABR assessment, and 26 were found with a hearing impairment, resulting in an incidence of HL 26/6034 = 4.3/1000. There were no risk factors in 9 (34.6%) of the Syrian newborns with HL, while 17 (65.4%) presented risk factors such as the number of days in the NICU, consanguineous marriage, hyperbilirubinemia and family history of HL.

### 3.15. Taiwan

Wu et al. [35] implemented a combined DPOAE + newborn genetic screening (NGS) program in 1017 infants at National Taiwan University Hospital. DPOAE was performed at 48 h and repeated before discharge, while blood samples were screened for GJB2, SLC26A4, and m.1555AG mutations. Infants with REFER OAE results or pathogenic genotypes underwent confirmatory ABR by three months. This dual-screening model successfully identified infants with subclinical or at-risk genetic variants, suggesting that integrating NGS with UNHS enhances the detection of progressive or late-onset HL.

### 3.16. Thailand

Five studies conducted in Thailand between 2005 and 2019 tested universal and targeted NHS programs, representing both tertiary and primary care settings.

Srisuparp et al. [36] evaluated a high-risk neonatal screening program at Siriraj Hospital in Bangkok, using an automated combined OAE/ABR device (AccuScreen) operated by trained nurses. Among 507 high-risk neonates, 6.7% (n = 34) had abnormal screening results, including 4.1% bilateral and 2.6% unilateral failures. Univariate analysis using a Chi-square test and multiple logistic regression analysis were used for identification of significant risk factors: independent risk factors for abnormal screening outcomes included craniofacial anomalies (≈42-fold) and mechanical ventilation > 5 days (≈4-fold). The median screening age was 19 days, and nearly all infants (97.3%) were screened before 3 months of age, confirming the feasibility of such a nurse-led program in resource-limited contexts.

Poonual et al. [37] implemented a three-step universal screening protocol at three tertiary hospitals in Northern Thailand using automated OAEs, conventional OAEs, and ABR. Among 3120 infants aged 3 months, 4.3% failed the OAE screening, and 5 (0.16%) were confirmed by ABR with a bilateral SNHL. Major risk factors for HL included LBW, low Apgar score, craniofacial anomalies, sepsis, and ototoxic drug exposure.

Poonual et al. [38] evaluated the outcomes of early identification and development of speech and auditory skills in the same cohort as in [37]. Of 3120 newborns screened by two-stage TEOAE + AABR, 7.5% failed the TEOAE and 4.3% failed the AABR test. At 6 months, 3.3% were diagnosed with congenital HL, though only 0.4% had permanent HL at 18 months. Early intervention before 6 months improved auditory and speech outcomes in all cases, though language development remained delayed in the majority. Major associated risk factors were craniofacial anomalies, ototoxic exposure, hyperbilirubinemia, low Apgar score, and sepsis.

On the same 3120 newborns of the two previous papers [37,38], Poonual et al. [39] proposed the COBRA score (by considering 5 risk factors: Craniofacial anomaly, ototoxicity, birth weight, relative history, Apgar score) as a simple clinical tool for predicting HL risk in primary care. Applied to the same Northern Thai cohort (n = 3120), the COBRA score, derived from multivariable Poisson risk regression, achieved an Area under ROC of 0.95, comparable to the JCIH risk criteria, and correctly identified all infants with confirmed HL (4.3% at 3 months by ABR). This approach was considered suitable for low-resource or primary care environments.

Pitathawatchai et al. [40] conducted a hospital-based program consisting of two stages of OAEs and one stage of ABR on a total of 6140 eligible newborns. Reported bilateral HL prevalence ranged between 0.2 and 0.3% (≈2–3 per 1000 births) with >80–90% follow-up completion, demonstrating high feasibility and national scalability. In total, 12 cases resulted in HL and were enrolled in an early intervention program. Nine of those infants presented normal speech development at 12 months. Data on the total costs of an UNHS program were also reported, with a total expenditure of 8944 USD for every infant diagnosed with sensorineural HL.

In summary, these five studies indicate that Thailand has successfully implemented multi-stage OAE/ABR-based NHS local programs since the early 2000s, achieving high coverage, early diagnosis, and improved outcomes with low false-positive rates. Integration of risk-based scoring systems such as COBRA could further enhance screening in peripheral health settings.

### 3.17. Turkey

Four studies conducted between 2007 and 2020 evaluated the implementation and performance of UNHS programs in Turkey, documenting a steady improvement in screening coverage, diagnostic accuracy, and follow-up adherence over time.

Tatli et al. [41] investigated the feasibility of NHS at a tertiary-level hospital in İzmir. The program used a two-stage protocol consisting of TEOAEs at 48 h postnatal age, followed by diagnostic ABR testing for infants who failed the initial screen. Out of 711 screened newborns, 1.2% (n = 9) were referred for diagnostic assessment, and 0.14% (1/711) were confirmed to have bilateral SNHL. The refer rate after the first TEOAE test was 3.7%, dropping to 1.2% after rescreening. The study demonstrated a 100% parental compliance for follow-up and concluded that NHS is feasible, rapid, and cost-effective even in non-university public hospitals. The authors emphasized that integration into routine postnatal care is key for national expansion.

Tasci et al. [42] conducted a large-scale prospective study in Ankara to evaluate the effectiveness of a two-stage OAE + ABR screening protocol across multiple hospitals. A total of 16,975 neonates were screened between January 2007 and February 2008.

The first-stage OAE REFER rate was 4.9%, reduced to 1.8% after repeat testing. Diagnostic ABR confirmed permanent congenital HL in 31 infants (0.18%), of which 22 (0.13%) were bilateral. The study identified LBW, NICU admission, and family history of HL as significant predictors of permanent HL. The authors concluded that a two-step OAE protocol followed by diagnostic ABR is an efficient, high-yield strategy for national implementation and recommended the creation of regional data registries to monitor screening performance.

Yılmazer et al. [43] analyzed 5985 newborns from two large hospitals in Istanbul (4111 healthy and 1874 high-risk infants) to assess follow-up outcomes and risk factors after newborn hearing screening. The program followed a two-phase model: all well babies (WB) were tested by TEOAEs, while ABR was used for the NICU residents or the WB REFER cases. The initial REFER rate was 4.2% among WB and 10.3% in the high-risk group. The overall hearing-loss prevalence was 1.8 per 1000 live births (11 infants total), of which 8 were bilateral. Parental compliance with diagnostic ABR follow-up was 87%. Among risk factors, prolonged NICU stay, hyperbilirubinemia, and ototoxic medication exposure were the strongest predictors of abnormal OAE results. The authors reported that integrating ABR into the second stage significantly reduced false-positive rates and increased the detection of auditory neuropathy cases. The study concluded that the Turkish national UNHS program had reached levels of quality comparable to those in European states, emphasizing the importance of standardized data recording and ongoing monitoring.

Kaplama et al. [34] in the previously mentioned paper on the Syrian infants, assessed data from 37,219 Turkish neonates with a two-stage TEOAE protocol followed, if necessary, by an AABR and in case of another REFER by a clinical ABR. In total, 132 infants were admitted to a clinical ABR assessment, and 84 were found with a hearing impairment, resulting in an incidence of HL 84/37,219 = 2.26/1000. There were no risk factors in 49 of the newborns with HL, while 35 (65.4%) presented risk factors such as the number of days in the NICU, hyperbilirubinemia, consanguineous marriage, and family history of HL.

Overall, these four Turkish studies highlight the progressive success of Turkey’s nationwide UNHS rollout, achieving screening coverage > 90%, follow-up rates > 85%, and a confirmed bilateral HL prevalence between 0.13% and 0.22%. The TEOAE + ABR two-stage protocol remains the national standard, demonstrating high diagnostic reliability, cost-effectiveness, and strong public compliance.

## 4. Discussion

The serious implications of a delayed detection of hearing impairment in the language development of the infant population have been presented and discussed in our previous reviews on NHS practices in Europe [8] and in Africa [9,10]. The objectives of this scoping review were the evaluation of the available NHS data in the Asian states and of any possible updates since the last reported estimates in 2020 by Neumann et al. [9]. In terms of updates, 15 out of the 31 papers used in this review were published from 2020 onwards. In Section 4.1, Section 4.2, Section 4.3, Section 4.4 and Section 4.5 will be reported data aggregated in five key areas targeting the five questions presented in the Introduction Section.

### 4.1. States Implementing NHS-UNHS Programs

Based on the reviewed evidence, UNHS programs have been implemented either nationally or regionally in multiple Asian and Middle Eastern states, including Indonesia, Iran, Israel, Jordan, Japan, Nepal, Philippines, Singapore, South Korea, Saudi Arabia, Taiwan, Thailand, and Turkey. The level of implementation varies widely: while states such as Israel, South Korea, Thailand, and Turkey have achieved a near-universal national coverage, others, including Indonesia, Jordan, and Nepal, are still developing regional or more often hospital-based screening initiatives. The latter was found to be the established starting-point for any NHS experience, as also observed in numerous African states [9,10].

### 4.2. Proportion of Newborns Screened

The reported NHS coverage ranged from 60 to 95%, with several national programs reporting near-complete participation. Iran and Israel achieved coverage above 94%, while Saudi Arabia, Thailand, and Turkey reported rates exceeding 90% of live births. In contrast, Indonesia and Nepal presented lower coverage levels, primarily due to resource limitations and decentralized health systems. Importantly, even in states with high coverage, disparities persist between urban and rural or low-resource areas, suggesting that infrastructure and workforce training remain essential to ensure equitable access. Several studies have reported screening on older infants aged 3 months [37] or even children [29].

The data on the follow-up rates (after the initial NHS start-up) varied considerably among the Asian states. Programs with centralized data systems and parental counselling, such as those in Iran, Israel, Saudi Arabia, and Turkey, achieved 85–95% follow-up compliance, comparable to European high-income states [6]. Conversely, LTF numbers remained a major challenge in the low-resource settings like Indonesia and Nepal, where LTF rates exceeded 10–15% in some reports. Consistent parental education, integration of screening data into national registries, and mandatory follow-up pathways were repeatedly identified as critical components for improving post-screening adherence.

### 4.3. Prevalence of Congenital and Bilateral HL

When reported, the prevalence of congenital HL ranged between 0.1% and 0.4% (1–4 per 1000 live births), values which align with the global NHS standards. Bilateral SNHL was consistently the predominant form, typically accounting for 70–90% of confirmed cases. High reported HL prevalences were seen in the datasets from Malaysia (15.3/1000), Singapore (15/1000) and Thailand (43/1000), but in order to achieve more objective estimates, much larger samples of neonates must be assessed. Notably, states with high rates of consanguineous marriages (Jordan, Saudi Arabia, and parts of Israel) exhibited increased prevalence and delayed-onset cases, underscoring the genetic contribution to pediatric HL in these populations.

### 4.4. Causes Leading to HL and Intervention Strategies

Very few studies have reported the probable causes of HL, including the standard risk factors (days in the NICU, ototoxic drugs, Low birth weight, family history, etc.) and the issue of consanguinity (which was also a significant factor in the reported African HL data).

A total of 3 studies (2 from North Korea and 1 from Taiwan) from the pool of 31 manuscripts provided information on Neonatal Genetic Screening, especially about mutations of the GJB2 and SLC26A4 genes. The reported findings are alarming since they show that infants passing the OAE + AABR tests can be positive for GJB2 or SLC26A4 mutations. These data confirm similar findings from Russia reported in a previous scoping review on the European data [6]. In this context, NHS protocols should be accompanied by an NGS to detect cases probably prone to progressive or late-onset HL.

### 4.5. Screening Protocols and OAE Technologies

Most states adopted the standard two- or three-stage screening models, by using in the first one- or two-stages the OAEs, followed by a clinical ABR for the OAE REFER cases or the assessment of NICU infants (in UNHS programs). This OAE-ABR combination minimized false-positive results and allowed detection of auditory neuropathy spectrum disorders.

Indonesia demonstrated that serial OAE testing at three time-points, at birth, at 1 month, and at 2 months, can effectively reduce the false referrals in resource-limited settings. Thailand and Turkey have standardized the use of a TEOAE + ABR protocol, while Japan and Taiwan additionally integrate genetic testing (e.g., GJB2, SLC26A4) to identify infants at risk for progressive or late-onset HL.

Emerging strategies, such as the COBRA risk score developed in Thailand, offer practical screening tools for primary care environments with limited technology and based on risk factors independently associated with HL.

### 4.6. Possible Limitations of the Study

We have identified three areas where the information from the current manuscript might be incomplete:

(i) As in our previous scoping reviews, some limitations might arise from the fact that available and probably significant screening data (for any state in question) are not reported in established impact factor sources and therefore cannot be utilized.
(ii) The pool of papers in this scoping review does not present sufficient information about the intervention strategies applied; once an HL case has been identified, therefore, it was not possible to report in depth any intervention activities. This aspect is quite common in the majority of programs and has also been noted in European and African NHS publications.
(iii) China and India were not included in the review because across these huge states, the sociopolitical gradient and the relative health policies change significantly. Our initial hypothesis was that these data could bias he overall Asian NHS report. A future manuscript will examine the NHS status of these two states.

### 4.7. Future Directions

From 2005 to 2025, OAEs have remained a cornerstone of UNHS across Asia. Programs that pair OAEs with rescreening, clear diagnostic referral pathways (AABR/ABR), and robust follow-up systems show better program performance [35]. Key priorities for the next decade are reducing the loss to follow-up index with digital registries, validating low-cost portable OAE devices, and producing multi-site comparative evidence to guide regionally appropriate protocols and policy.

## 5. Conclusions

The data from the literature indicate that many Asian middle-income states have successfully transitioned from pilot hospital-based projects to sustainable UNHS programs, achieving international standards. The overall evidence supports the feasibility and cost-effectiveness of UNHS programs across diverse socioeconomic contexts, provided that screening is accompanied by consistent follow-up, parent engagement, and appropriate referral systems. Fragmented database systems (primarily from local NHS experiences) seem to delay a more nationwide NHS organization.

## Figures and Tables

**Table 1:** The 31 eligible papers after the filtering process are as follows. The data are presented in alphabetical order for each state; NA = Not Available Information; NICU, Neonatal Intensive Care Unit; NHS, Neonatal Hearing Screening).

n	State	Region/City or Town	Sample Size (n)	Study Period	Authors (First Name)	Publication Year
1	Indonesia	Surabaya	60	2023	Falerina [13]	2023
2	Iran	Southwestern	92,521	March 2013–April 2016	Saki [14]	2017
3	Israel	Ziv Medical Center	5496 (5212)	Mar 2010–Dec 2011	Gilbey [15]	2013
4		Beer-Sheva	238	2024	Ziv [16]	2024
5		NA	5621	2014–2023	Farladansky-Gershnabel [17]	2025
6	Japan	Okayama	47,346	2001–2005	Fukushima [18]	2008
7		Akita	35,461	2012–2016	Sato [19]	2020
8	Jordan	Amman	416	2024	Qanawati [20]	2024
9	Malaysia	Kuala	2713 (NICU)	2014–2016	Abdullah [21]	2020
10		Kuala, Sarawak, Kedah, Perak	58,862 (28,432 + 30,340)	2015–2016	Wong [22]	2021
11	Nepal	Kathmandu	5517	2015–2016	Shrestha [23]	2017
12	Oman	Al-Seeb	12,743	2016–2018	Kolethekkat [24]	2020
13	Pakistan	Karachi	267	2020–2021	Pyarali [25]	2023
14	Philippines	Manila	NA	2009 -	Labra [26]	2023
15	Saudi Arabia	Jeddah	11,986	1996–2004	Habib [27]	2005
16		Riyadh	20,171	2016	Alanazi [28]	2020
17		Riyadh	130 (children)	2025	Elbeltagy [29]	2025
18	Singapore	Singapore	100,225	2002–2009	Jayagobi [30]	2019
19		Singapore	29,972	2004–2014	Tang [31]	2022
20	South Korea	Seoul	43	NA	Kim [32]	2013
21		Seoul	185 children (109 NHS data)	2008–2017	Lee [33]	2019
22	Syria	Refugees in Turkey	6034	2018	Kaplama [34]	2020
23	Taiwan	Taiwan	1017	2011	Wu [35]	2011
24	Thailand	Bangkok	507 high-risk infants	2005 (18-month study)	Srisuparp [36]	2005
25		Northern	3120 (infants at 3 months)	Nov 2010–May 2012	Poonual [37]	2015
26		Northern	3120 (development evaluation from 6 to 18 months)	Nov 2010–May 2012	Poonual [38]	2017a
27		Northern	3120	Nov 2010–May 2012	Poonual [39]	2017b
28		Southern	6140	January–July 2017	Pitathawatchai [40]	2019
29	Turkey	Izmir	711	2007	Tatli [41]	2007
30		Ankara	16,975	Jan 2007–Feb 2008	Tasci [42]	2010
31		Istanbul	4111 healthy; 1874 high-risk	2016	Yılmazer [43]	2016
32		Sanliurfa	37,219	01–12/2018	Kaplama [34]	2020

**Table 2:** Data presenting the various Asian NHS national activities. The national data are presented in alphabetical order. The second column presents the NHS protocols used. The third column reports the overall HL prevalence, or the bilateral and unilateral HL estimates, when calculated or reported. The fourth column reports the major causes of HL as reported by the corresponding publication. The last two columns refer to the first author and the year of the relevant publication. Abbreviations: OAEs: Otoacoustic Emissions; TEOAE: Transient-Evoked OAE; DPOAE: Distortion Product OAE; AABR: Automated Auditory Brainstem Response; ABR: Auditory Brainstem Response; ASSR: Auditory Steady State Response; SNHL: Sensorineural Hearing Loss; CHL: Conductive Hearing Loss; MHL: Mixed Hearing Loss; CI: Cochlear Implant; HL: Hearing Loss; LBW: Low Birth Weight; UNHS: Universal Newborn Hearing Screening; PTA: Pure Tone Average; bil. HL: Bilateral HL; uni. HL: Unilateral HL. #: sample is too small to generalize; NA = Not Available Information.

State	Screening Protocol (OAE/ABR)	Hearing Loss Prevalence	Causes/Risk Factors	Author (First)	Year
Indonesia	DPOAE at >24 h, 1 and 2 months; ABR for persistent REFER cases	2/60 #	Prematurity, LBW, low Apgar; most improved after serial testing	Falerina [13]	2023
Iran	Two-stage TEOAE + AABR; diagnostic ABR/OAE at ≤3 mo	bil. HL (1.52/1000) uni. HL (0.89/1000)	87% sensorineural, 12.5% auditory neuropathy; higher rural referral	Saki [14]	2017
Israel	TEOAE + AABR; high-risk infants	7/5212 1.3/1000 Estimate is optimistic since, for significant number of REFERs, testing was not possible	NA	Gilbey [15]	2013
	OAE + ABR (UNHS); post-UNHS cohort analysis	238 children with confirmed HL Initial sample size is NOT reported	Consanguinity is associated with higher bilateral HL and delayed/poor CI compliance	Ziv [16]	2024
	TEOAE within 48–72 h after birth	NA	Cesarean section and vacuum-assisted deliveries increased false-positive risk (OR 7–15×)	Farladansky-Gershnabel [17]	2025
Japan	Two-stage AABR + clinical ABR follow-up for 24 months	bil. HL (108/47,346) uni. HL (140/47,346) 5.2/1000	NA	Fukushima [18]	2008
	Two-stage AABR + clinical ABR or ASSR	bil. HL (47/33,547) uni. HL (97/33,547) 4.3/1000	For 1 bil. HL cases, mutation of Gap-junction beta-2 protein was found	Sato [19]	2020
Jordan	DPOAE + ABR	77.9% with HL (65% SNHL; 33% CHL; 1% MHL) #	Strong association with consanguineous marriage (38.46%) and family history	Qanawati [20]	2024
Malaysia	OAE (not specified) + AABR + clinical ABR	15.36/1000	Craniofacial anomalies were the factor most related to HL; Infants with 3 or more risk factors presented 100% HL	Abdullah [21]	2020
	DPOAE+ AABR	3.15/1000	NA	Wong [22]	2021
Nepal	Two-stage ABR (birth + 6 weeks); diagnostic ABR for persistent REFER	Apparent 6.7% (374/5517 referred); higher among NICU infants (9%) #	Risk factors: LBW, prematurity, ototoxic drugs, hyperbilirubinemia, mechanical ventilation	Shrestha [23]	2017
Oman	Two-stage TEOEA + Clinical ABR	3.37/1000	Craniofacial complications and syndromic HL	Kolethekkat [24]	2020
Pakistan	Two-OAE stages (protocol not specified) + clinical ABR.	18/267 HL #	Gestational age, craniofacial abnormalities	Pyarali [25]	2023
Philippines	Two-stage testing + Clinical ABR/ASSR	NA	NA	Labra [26]	2016
Saudi Arabia	Two-stage TEOAE (<48 h + day 5) + diagnostic ABR	0.18% HL; 0.17% bil. SNHL	Congenital bil. SNHL dominant (20/22)	Habib [27]	2005
	PTA + TEOAE + Tympanometry (school-age follow-up)	0.17–0.18% congenital bil. HL; 5.4% prevalence of delayed onset	Consanguinity increases risk of late SNHL	Alanazi [28]	2025
	Two-stage TEOAE (<48 h, <2 weeks)	referral rate: 1.33%	NA	Elbeltagy [29]	2020
Singapore	Two-stage AABR	35/1000 overall 19.9/1000 permanent HL	Craniofacial anomalies, mechanical ventilation > 5 days auditory neuropathy incidence 1.1/1000	Jayagobi [30]	2020
	Two-stage TEOAE+ AABR	117/29,671 or 3.9/1000	NA	Tang [31]	2023
South Korea	ABR (majority), TEOAE (minority); confirmatory ABR/ASSR	NA	Biallelic SLC26A4 (PDS) mutations; late diagnosis	Kim [32]	2013
	TEOAE + ABR; In case of REFER confirmation by ABR + ASSR	NA	SLC26A4 (PDS) mutations (41% in NHS-pass group); later diagnosis in NHS-referred group	Lee [33]	2009
Syria	Two-stage TEOAE + AABR	26/6034 or 4.3/1000.	17 (65.4%) presented risk factors as n days NICU stay, hyperbilirubinemia, consanguineous marriage, and family history of HL.	Kaplama [34]	2020
Taiwan	Two-stage DPOAE + genetic screening (GJB2, SLC26A4, m.1555A > G) + ABR	Hearing screening referral rate: 3.7%	Genetic causes could not be identified by UNHL	Wu [35]	2011
Thailand	Automated OAE/ABR (AccuScreen) in high-risk infants	Bil. HL 4.1% uni. HL 2.6% 6.7/1000	Risk factors: craniofacial anomalies, mechanical ventilation > 5 days	Srisuparp [36]	2005
	Two-stage TEOAE + ABR (prospective cohort study, infant at 3 months)	135/3120 or 43/1000 (very high value)	Risk factors independently associated with bilateral HL (birth weight, craniofacial anomalies, sepsis and ototoxic exposure)	Poonual [37]	2015
	Evaluation following the Two-stage TEOAE + ABR from 6 to 18 months (analytic prospective study)	14/3120 or 4.48/1000	Risk factors: ototoxic exposure, low APGAR, craniofacial anomalies (see details in Table 5 of [38])	Poonual [38]	2017a
	Two-stage TEOAE + ABR; risk prediction via COBRA score	43/1000	Risk factors COBRA score: (craniofacial anomaly, ototoxicity, birth weight, relative history, Apgar); 80% of existing HR was detected	Poonual [39]	2017b
	OAE (TEOAE) two-stage + ABR; confirmation speech development at 12 months	12/5859 or 2.04/1000	Assisted ventilation, ototoxic drugs, duration in NICU, syndromic HL	Pitathawatchai [40]	2019
Turkey	Two-stage TEOAE + ABR (diagnostic)	1.21/1000	SNHL predominant	Tatli [41]	2007
	Two-stage TEOAE + ABR (diagnostic)	bil. HL (28/16,975), uni. HL (10/16,975) 2.2/1000	Ototoxic drugs (39.5%), NICU stay > 7 days, prematurity (39.5%)	Tasci [42]	2010
	Two-stage TEOAE + ABR (diagnostic)	bil. HL (8/5985) uni. HL (3/5985) 1.8/1000	(60%) prolonged mechanical ventilation or neonatal intensive care, (50%) consanguineous marriage, (10%) LBW, (10%) family history of HI and (10%) hyperbilirubinemia.	Yılmazer [43]	2016
	Two-stage TEOAE + AABR	84/37,219 or 2.26/1000.	35 (65.4%) risk factors such as n days NICU stay, hyperbilirubinemia, consanguineous marriage, family history of HL	Kaplama [34]	2020

## Data Availability

The original contributions presented in this study are included in the article. Further inquiries can be directed to the corresponding author.

## Supplementary Materials

The following supporting information can be downloaded at: https://www.mdpi.com/article/10.3390/children13010060/s1, Table S1: PRISMA 2020 Checklist [46].

## Abbreviations

AABR	Automated Auditory Brainstem Response
ABR	Auditory Brainstem Response
ASSR	Auditory Steady State Response
BD	Bilateral Deafness
CHL	Conductive Hearing Loss
CI	Cochlear Implant
DPOAE	Distortion Product OAE
EDHI	Early Hearing Detection and Intervention
ENT	Ear, Nose, and Throat
HL	Hearing Loss
LBW	Low Birth Weight
LTF	Loss to Follow-Up
MHL	Mixed Hearing Loss
NHS	Neonatal Hearing Screening
NICU	Neonatal Intensive Care Unit
OAEs	Otoacoustic Emissions
PTA	Pure Tone Average
SNHL	Sensorineural Hearing Loss
TEOAE	Transient-Evoked OAE
UNHS	Universal Neonatal Hearing Screening

## Appendix A

The Asian continent is composed of 50 states. In various contexts, Asia is divided into five subregions (Central, Eastern, Southern, Southeastern, and Western Asia) [12]. In this review, we have considered the whole continent without any specific geographical divisions. Asia accounts for approximately 59–60% of the world’s population and about 30% of the Earth’s total land area.

**Table A1:** Assessed states (in bold) for the presence of NHS activities. Data from China and India (in shaded cells) were not considered in the review.

ID	Country	Population	Studies Selected	Region
1	Afghanistan	43,844,111	-	South Asia
2	Armenia	2,952,365	-	Western Asia
3	Azerbaijan	10,397,713	-	Western Asia
4	Bahrain	1,643,332	-	Western Asia
5	Bangladesh	175,686,899	-	South Asia
6	Bhutan	796,682	-	South Asia
7	Brunei	466,330	-	Southeast Asia
8	Cambodia	17,847,982	-	Southeast Asia
9	China	1,416,096,094	-	East Asia
10	China Hong Kong		-	East Asia
11	China Macao			East Asia
12	Cyprus	1,370,754	-	Western Asia
13	Georgia	3,806,671	-	Western Asia
14	India	1,463,865,525	-	South Asia
**15**	**Indonesia**	**285,721,236**	**1**	**Southeast Asia**
**16**	**Iran**	**92,417,681**	**1**	**South Asia**
17	Iraq	47,020,774	-	Western Asia
**18**	**Israel**	**9,517,181**	**3**	**Western Asia**
**19**	**Japan**	**123,103,479**	**2**	**East Asia**
**20**	**Jordan**	**11,520,684**	**1**	**Western Asia**
21	Kazakhstan	20,843,754	-	Central Asia
22	Kuwait	5,026,078	-	Western Asia
23	Kyrgyzstan	7,295,034	-	Central Asia
24	Laos	7,873,046	-	Southeast Asia
25	Lebanon	5,849,421	-	Western Asia
**26**	**Malaysia**	**35,977,838**	**2**	**Southeast Asia**
27	Maldives	529,676	-	South Asia
28	Mongolia	3,517,100	-	East Asia
29	Myanmar	54,850,648	-	Southeast Asia
**30**	**Nepal**	**29,618,118**	**1**	**South Asia**
31	North Korea	26,571,036	-	East Asia
**32**	**Oman**	**5,494,691**	**1**	**Western Asia**
**33**	**Pakistan**	**255,219,554**	**1**	**South Asia**
34	Palestine	5,589,623	-	Western Asia
**35**	**Philippines**	**116,786,962**	**1**	**Southeast Asia**
36	Qatar	3,115,889	-	Western Asia
**37**	**Saudi Arabia**	**34,566,328**	**3**	**Western Asia**
**38**	**Singapore**	**5,870,750**	**2**	**Southeast Asia**
**39**	**South Korea**	**51,667,029**	**2**	**East Asia**
40	Sri Lanka	23,229,470	-	South Asia
**41**	**Syria**	**25,620,427**	**1**	**Western Asia**
42	Tajikistan	10,786,734	-	Central Asia
**43**	**Thailand**	**71,619,863**	**5**	**Southeast Asia**
44	Timor–Leste	1,418,517	-	Southeast Asia
**45**	**Turkey**	**87,685,426**	**3**	**Western Asia**
46	Turkmenistan	7,618,847	-	Central Asia
**47**	United Arab Emirates	11,346,000	-	Western Asia
48	Uzbekistan	37,053,428	-	Central Asia
49	Vietnam	101,598,527	-	Southeast Asia
50	Yemen	41,773,878	-	Western Asia
	**Taiwan ***	**23,073,123**	**1**	**East Asia**
Selected	17 *	1,253,753,247	31	7 W; 5SE; 3S; 3E

The regional classifications which were used in this paper were taken from [12] https://unstats.un.org/unsd/methodology/m49/ (accessed on 4 July 2025)]: *East Asia*: China, China Macao, China Hong Kong, Japan, Mongolia, North Korea, Korea. *Southeast Asia:* Brunei, Cambodia, Indonesia, Laos, Malaysia, Myanmar, Philippines, Singapore, Thailand, Timor–Leste, Vietnam. *South Asia*: Afghanistan, Bangladesh, Bhutan, India, Iran, Maldives, Nepal, Pakistan, Sri Lanka. *Western Asia:* Armenia, Azerbaijan, Bahrain, Cyprus, Georgia, Iraq, Israel, Jordan, Kuwait, Lebanon, Oman, Palestine, Qatar, Saudi Arabia, Syria, Turkey, United Arab Emirates, Yemen. *Central Asia:* Kazakhstan, Kyrgyzstan, Tajikistan, Turkmenistan, Uzbekistan. (* Taiwan is not included in 50 by [12]). We selected 31 papers from 18 states (in bold in Table A1), focusing our investigation on approximately 65% (≈1,253,753,247) of the total Asian population without China and India (≈1,924,127,566).
